# Cutaneous melanocytic tumor with CRTC1::TRIM11 fusion in an adolescent: A case report and focused literature review of reported pediatric cases

**DOI:** 10.1016/j.jdcr.2026.05.033

**Published:** 2026-05-22

**Authors:** Coral Martes-Villalobos, Sri Naidnur, Valeria González-Molina, Sujitha Yadlapati, Rick Lin, Emily DeSantis

**Affiliations:** a Universidad Central del Caribe School of Medicine, Bayamon, PR; b Boston University Chobanian & Avedisian School of Medicine, Boston, Massachusetts; c Oasis Dermatology Group, McAllen, Texas; d HCA Healthcare Corpus Christi Medical Center-Bay Area Dermatology Residency Program, McAllen, Texas; e University of North Carolina, Chapel Hill, North Carolina; f Dermatopathology Department at Sagis Diagnostics, Houston, Texas

**Keywords:** CRTC1::TRIM11 translocation, cutaneous melanocytic tumor with CRTC1::TRIM11 fusion, cutaneous melanocytoma, gene fusion tumor, melanocytic neoplasm, pediatric melanocytic tumor

## Introduction

Melanocytic tumors of the skin encompass a broad spectrum of entities and may present significant diagnostic challenges. Although most melanocytic neoplasms are classified based on morphologic, immunohistochemical (IHC), and molecular features, some lesions demonstrate ambiguous characteristics that defy conventional categorization. Cutaneous melanocytic tumor with CREB-regulated transcription coactivator 1–tripartite motif-containing 11 fusion (CRTC1::TRIM11) is a recently described neoplasm now recognized as a distinct diagnostic entity that must be distinguished from its histologic mimics.^1^

Initially described in 2018 as “cutaneous melanocytoma with CRTC1::TRIM11 fusion,” the terminology evolved to cutaneous melanocytic tumor (CMTCT) following recognition of occasional aggressive behavior.^1^, ^2^, ^3^ Although more than 50 cases have been reported overall, the vast majority involve adults, with pediatric cases remaining rare.^2^ To the best of our knowledge, 9 pediatric cases of CMTCT (≤18 years) have been reported to date. Herein, we report the 10th case and present a focused literature review.

## Case report

**Fig 1:**
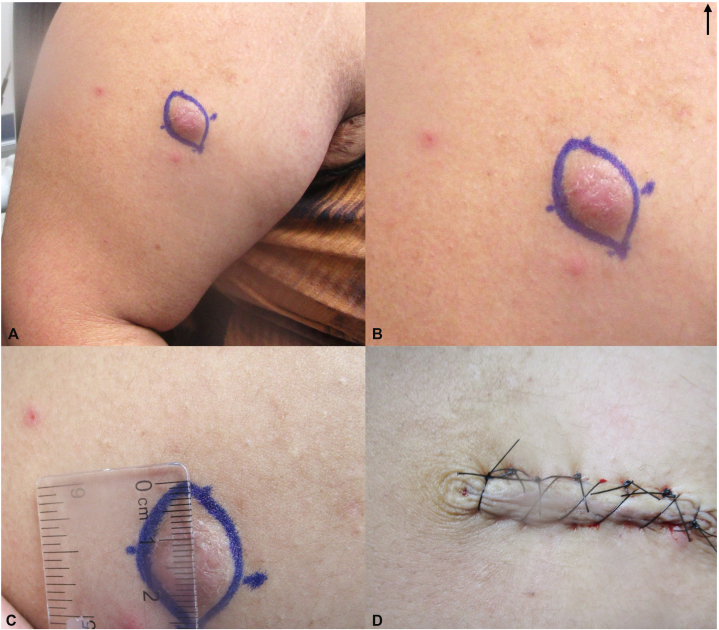
Clinical image of CMTCT demonstrating a well-defined, 2 × 2 cm light-brown to skin-colored nodule, marked prior to excisional biopsy **(A-C)**. A clinical image of the sutures performed after excision of the nodule **(D)**.

A 17-year-old female presented with a slow-growing, non-tender lesion on the right upper arm that had been present for over a year. Physical examination revealed a well-defined 2 × 2 cm light brown to skin-colored nodule, which was subsequently excised (Fig 1, *A*-*D*). The clinical differential diagnosis included amelanotic melanoma, atypical Spitz nevus, dermatofibrosarcoma protuberans, and clear cell sarcoma.

**Fig 2:**
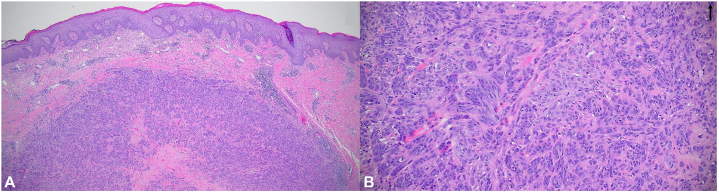
Low-power view demonstrating a well-circumscribed nodule within the dermis **(A)**. At higher magnification, the cells demonstrate round-to-ovoid, vesicular nuclei with small nucleoli and moderate light pink cytoplasm. Scattered mitotic figures are present (H&E, ×20) **(B)**.

**Fig 3:**
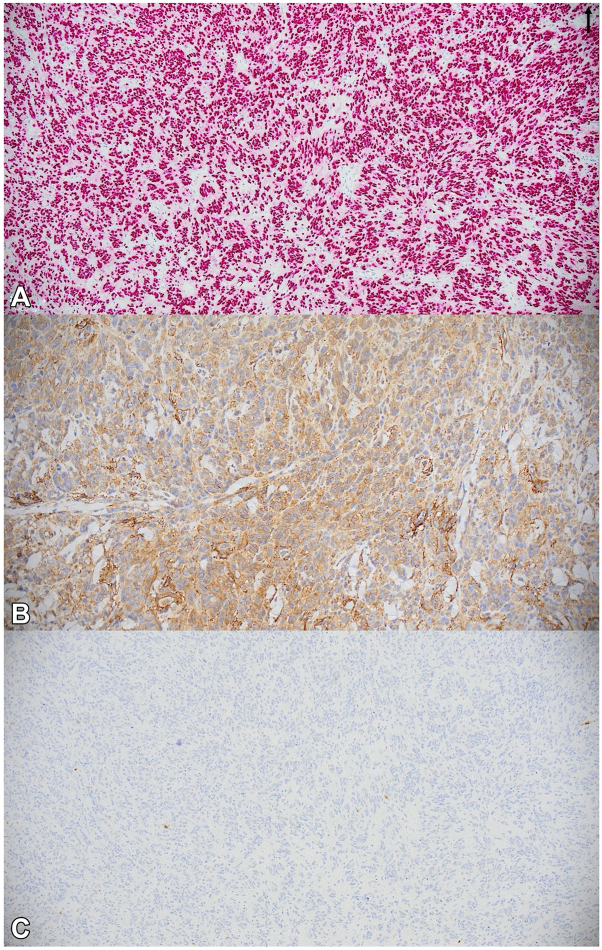
An immunohistochemical stain for SOX-10 shows strong nuclear positivity on 10× magnification **(A)**. The tumor demonstrates diffuse cytoplasmic staining with CD99 **(B)**. No S100 expression seen **(C)**.

Excisional biopsy revealed a well-circumscribed dermal nodule composed of nests and fascicles of cells with epithelioid to plump spindle morphology (Fig 2, *A*). Central necrosis and numerous mitotic figures were present. Tumor cells exhibited vesicular, round-to-ovoid nuclei, small nucleoli, and light eosinophilic cytoplasm (Fig 2, *B*). Immunohistochemistry showed positivity for SOX10 (Fig 3, *A*), CD99 (Fig 3, *B*), and FLI-1, with retained INI-1 expression. Stains for HMB-45, MART-1, S100 (Fig 3, *C*), EMA, cytokeratins (AE1/AE3, CK8/18, CK7, CK20), p63, and CD34 were negative, supporting a distinct entity rather than conventional melanoma or sarcoma.

Next-generation sequencing identified a CRTC1 exon 1–TRIM11 exon 2 fusion, confirming the diagnosis of CMTCT. The patient underwent wide local excision with subsequent re-excision to achieve negative margins. Sentinel lymph node biopsy and imaging were negative for metastasis. The patient was subsequently lost to follow-up.

## Discussion

CMTCT is a rare melanocytic neoplasm that typically presents as a slow-growing, skin-colored nodule involving the extremities or trunk, and less commonly the head, neck, or mucosa.^2^ Pediatric cases most often arise on distal extremities and demonstrate a slight female predominance, consistent with our review (Table I).^2^^,^^3^

**Table I:** Pediatric and adolescent (≤18 years) CRTC1::TRIM11 fusion cutaneous melanocytic tumor (CMTCT) cases^3^, ^4^, ^5^, ^6^, ^7^, ^8^

Section 1. Clinical and outcome characteristics										
Case	Age	Sex	Location	Treatment	Fusion	Recurrence	SLN biopsy	Metastasis	Follow-up	Reference
1	5 y	Female	Right arm	Excision; pembrolizumab	Positive	Yes	Performed	Yes	19 mo; metastatic	Vest et al^5^^,^^6^
2	11 y	Female	Lower leg	Excision	Positive	No	–	–	10 mo	Ko et al^4^
3	11 y	–	–	–	Positive	–	–	–	–	Hanna et al^3^
4	12 y	–	–	–	Positive	–	–	–	–	Hanna et al^3^
5	14 y	Female	Right ear	Excision	Positive	–	Performed	–	39 mo	Sargen et al^9^
6	16 y	Male	Right forearm	Excision × 2	Positive	–	–	–	Lost to follow up	Sargen et al^9^
7	17 y	Female	Upper back	Excision + systemic therapy∗	Positive	Yes	Performed	Yes	18 mo (lethal)	Beatson et al^7^
8	17 y	Male	Right ankle	Excision	–	–	Performed	Popliteal LN metastasis	48 mon, metastatic	Sargen et al^9^
9	**17 y**	**Female**	**Right upper arm**	**Excision and re-excision**	**Positive**	**–**	**Performed**	**–**	**Lost to follow-up**	**This case (2026)**
10	18 y	Female	Left leg	Excision and re-excision	Positive	No	–	–	6 mo	Duan et al^8^

Section 2. Most relevant immunohistochemical findings^3^, ^4^, ^5^, ^6^, ^7^, ^8^										
Case	Age	SOX10	MITF	S100	MelanA	HMB-45	PRAME	MART1	p16	p63
1	5 y	Positive	–	Positive	Negative	–	–	–	Negative	–
2	11 y	Positive	–	Negative	Focal+	Focal+	–	Focal +	–	Negative
3	11 y	–	–	–	–	–	–	–	–	–
4	12 y	–	–	–	–	–	–	–	–	–
5	14 y	Positive	–	–	Focal+	–	–	–	–	–
6	16 y	Positive	–	–	–	–	–	–	Negative	–
7	17 y	Positive	–	Positive	Focal+	–	Negative	–	–	–
8	17 y	Positive	–	–	Negative	–	–	–	Negative	–
**9**	**17 y**	**Positive**	**–**	**Negative**	**–**	**Negative**	**–**	**Negative**	**–**	**Negative**
10	18 y	Positive	–	Positive	Positive	Focal+	Positive	–	–	–

Table I summarizes the reported pediatric and adolescent (≤18 years) cases of CMTCT, including the present case. Clinical characteristics, treatments, and outcomes (recurrence, SLN biopsy, metastasis, and follow-up) are presented alongside immunohistochemical findings for each case, highlighting the variable clinical behavior and heterogeneous marker expression in this population.

‘–’ indicates data not specified or not performed in the original report.

*SLN*, Sentinel lymph node.

∗ Systemic therapies included nivolumab/relatlimab, cisplatin, vinblastine, dacarbazine (CVD), cabozantinib/nivolumab, lifileucel, venetoclax, and encorafenib/binimetinib.

## Microscopic features of CMTCT

• Presents as a sharply circumscribed dermal or subcutaneous proliferation composed of epithelioid and spindle cells arranged in nests or short fascicles within a collagenous stroma.^1^
• Cytologic atypia is moderate, with occasional nuclear pseudoinclusions.
• Mitotic figures and necrosis are generally reported as uncommon in prior studies; however, both features may be present.
• Epidermal involvement and marked pleomorphism are usually absent.^1^^,^^4^

## Immunohistochemical features of CMTCT

• Often expresses SOX10, whereas S100, HMB-45, and MART-1 expression is variable.^4^
• A high Ki-67 proliferation index has been reported in select cases.^3^
• Positive CD99 and FLI-1 expression has been reported, which may mimic Ewing sarcoma and warrant further molecular workup.

## Diagnosis

• Definitive diagnosis relies on integrated clinicopathologic, immunohistochemical, and molecular correlation, as well as confirmation of the pathognomonic CRTC1::TRIM11 gene fusion.^1^, ^2^, ^3^, ^4^

## Prognosis and management

• While most cases follow an indolent course, a subset demonstrates unpredictable behavior, including recurrence and metastasis, as evidenced in cases 1, 7, and 8 in Tables I.^3^, ^4^, ^5^, ^6^
• Three pediatric cases highlight this potential: a 5-year-old who developed nodal and pulmonary metastases 19 months after diagnosis, with imaging showing interval right arm/axillary lymph node enlargement prompting biopsies (case 1); a 17-year-old had micrometastasis of popliteal lymph nodes (case 8), and another 17-year-old patient experienced metastatic progression with a fatal outcome despite multimodal therapy (case 7).^3^, ^4^, ^5^, ^6^
• In some cases, sentinel lymph node biopsy was not performed at the time of initial diagnosis but was later pursued during follow-up, underscoring the importance of continued clinical surveillance and reassessment over time.^5^^,^^6^
• These findings highlight the need for long-term surveillance in pediatric patients, even when initial clinical behavior appears indolent, and excision margins are clear.
• Accordingly, sentinel lymph node evaluation was included in Table I, when available, to underscore the relevance of thorough regional lymph node assessment during clinical follow-up, including palpation, and to note that, in select cases with clinicopathologic uncertainty, sentinel lymph node biopsy may be considered to support management decisions.
• The evolving recognition of delayed metastasis in cases initially considered low risk reinforces the need for careful longitudinal monitoring and continued reporting of pediatric cases to better define prognostic factors and biologic behavior in this rare entity.

## Focused pediatric literature review

• The comparative synthesis provided in Table I highlights this variability and offers a focused overview of reported pediatric cases. Section 1 summarizes demographic, clinical, and outcome data, while Section 2 highlights the heterogeneity of immunohistochemical staining patterns.
• This side-by-side comparison emphasizes the educational value of cumulative reporting and may assist clinicians and pathologists when evaluating diagnostically ambiguous lesions in younger patients.
• The table consolidates, to the best of our knowledge, reported pediatric cases in the literature.
• Continued reporting and careful characterization of pediatric cases will be essential to refine risk stratification and guide management of this rare entity.

### Declaration of generative AI and AI-assisted technologies in the writing process

During the preparation of this work, the authors used ChatGPT and Grammarly only for language editing. After using this tool, the authors reviewed and edited the content as needed and take full responsibility for the content of the publication.

## Conflicts of interest

None disclosed.
